# Implementation of the multicountry WHO COVID-19 pregnancy cohort study: challenges and lessons learned during the pandemic

**DOI:** 10.1186/s12978-025-02080-4

**Published:** 2025-11-12

**Authors:** Maria Laura Costa, Renato Teixeira Souza, Jose Guilherme Cecatti, Sami L. Gottlieb, Marie Delnord, Soe Soe Thwin, Ndema Abu Habib, Ronaldo Silva, Daniel Giordano, Anna Thorson, Nathalie J. Broutet, Edgardo Abalos, Seni Kouanda, Kwasi Torpey, Emefa Modey, Erlidia Llamas-Clark, Sarah Saleem, Saleem Jessani, Marleen Temmerman, Ingrid Gichere, Beth Maina, Henda Triki, Mariem Gdoura, Ibukun-Oluwa Omolade Abejirinde, Alejandro Orrico-Sánchez, Sergio Munoz, Edna Kara

**Affiliations:** 1 Department of Obstetrics and Gynecology, University of Campinas, Campjnas, São Paulo Brazil; 2 UNDP/UNFPA/UNICEF/WHO/World Bank Special Programme of Research, Development and Research Training in Human Reproduction (HRP), Department of Sexual and Reproductive Health and Research, World Health Organization, Geneva, Switzerland; 3 Centro Rosarino de Estudios Perinatales (CREP), Rosario, Argentina; 4 Institut de Recherche en Sciences de la Santé (IRSS), Ouagadougou, Burkina Faso; 5 School of Public Health, University of Ghana, Accra, Ghana; 6 Department of Obstetrics and Gynecology, College of Medicine, University of the Philippines, Manila, Philippines and Institute of Child Health and Human Development, National Institutes of Health, University of the Philippines, Manila, Philippines; 7 The Aga Khan University, Karachi, Pakistan; 8 Aga Khan University, Nairobi, Kenya; 9 Institut Pasteur de Tunis, Tunis, Tunisia; 10 Dalla Lana School of Public Health, University of Toronto, Toronto, Canada; 11 HealthPro Research and Consultancy, Toronto, Canada; 12 Vaccine Research Department of Fisabio-Public Health and Hospital Dr. Peset of Valencia, Valencia, Spain; 13 University de La Frontera, Temuco, Chile

## Abstract

**Introduction:**

A generic research protocol was developed for a prospective cohort study to allow systematic, harmonized data collection of the impact of SARS-CoV-2 infection and vaccination during pregnancy on maternal, obstetric, and neonatal outcomes across different settings. This article describes the study conception, development, implementation, challenges, and key lessons learned within study sites across the world.

**Methods:**

The protocol was implemented in 43 facilities in 10 countries during the pandemic, involving consecutive recruitment of over 16,000 pregnant or postpartum women. We evaluated selection of study sites, ethical approvals, staff recruitment and training, recruitment and follow-up, and incorporation of new elements over the course of the pandemic across the study sites.

**Results:**

Study implementation in multiple LMIC settings was feasible; however, major challenges included delays in study implementation due to ethical approval procedures and availability of testing for exposure assessment. Implementation of research during a constantly evolving pandemic context led to the need for amended protocols, adjusted sample sizes, new outcomes and variables, repeated review by the Ethical Committees and adapted laboratory protocols. For example, the first COVID-19 vaccines became available after the study had started, with the need to modify the data collection forms and serologic testing algorithm to allow incorporation of this information in the study structure and analysis.

**Conclusion:**

Study implementation during a pandemic in different countries and periods was challenging but is not only expected to provide important information on the effects of SARS-CoV-2 infection and vaccination on pregnancy, but also on conducting research during future outbreaks. More streamlined ethics reviews during pandemics, availability of generic protocols in advance, and sites in LMICs ready to activate in an outbreak, as opposed to triggering processes during a crisis, would be highly beneficial.

## What is already known on this topic

- During the COVID-19 pandemic, SARS-CoV-2 infection during pregnancy was associated with increased risk for adverse maternal and perinatal outcomes;
- WHO had demonstrated during previous outbreaks the importance of having harmonized data across countries for pooling and meta-analyses;

What this study adds

- Understanding challenges in implementing a research protocol during a pandemic in low- and middle-income countries (LMICs) is key to planning for future healthcare crises;
- Lessons learned include the need for more streamlined ethics reviews during pandemics, availability of generic pregnancy-specific protocols in advance, and sites in LMICs ready to activate in an outbreak, as opposed to triggering the processes during a crisis;

How this study might affect research, practice or policy

- The details of study project implementation are rarely published. Sharing the challenges and lessons learned in implementing such a major protocol, with its combination of clinical data collection, viral investigation and diverse sample collection in different LMICs, is an opportunity to inform similar studies and provide tools for future endeavours.

## Introduction

During the COVID-19 pandemic, pregnancy and the postpartum period were conditions associated with increased risk for adverse outcomes, with increased stillbirth and maternal mortality reported early in the pandemic [1]. Severe acute respiratory coronavirus-2 (SARS-CoV-2) infection during pregnancy has also been linked with preterm birth, low birth weight, and increased neonatal intensive care unit (NICU) admission. However, data regarding the effect of COVID-19 on pregnancy specifically in low-and middle-income countries (LMICs) has been under-represented (2). Delays in healthcare and challenges in providing appropriate care and vaccination need to be considered in understanding how COVID-19-related adverse outcomes contribute to the global burden of the disease in these settings [2, 3].

During the initial stages of the pandemic, the World Health Organization (WHO) developed a generic research protocol for a prospective cohort study to allow systematic and harmonized data collection on the impact of SARS-CoV-2 infection during pregnancy (available at *Generic protocol: a prospective cohort study investigating maternal*,* pregnancy and neonatal outcomes for women and neonates infected with SARS-CoV-2*,* 1 November 2022 (who.int)*). The aim of the study was to investigate how SARS-CoV-2 infection during pregnancy affects maternal, pregnancy, neonatal and postpartum outcomes in women and their neonates. The protocol was designed to be conducted in different settings, especially LMICs, and adapted to each study site according to resource availability and local conditions, in a format that would facilitate pooling of data to increase power for analysing even uncommon outcomes.

The WHO cohort study was implemented in 10 countries, comprising 43 different maternity hospitals, with implementation starting in the first sites in February 2021. The study also was dynamically adapted to changes imposed by the pandemic and its management, especially the need to consider the impact of COVID-19 vaccination among pregnant women, with necessary amendments for new objectives, sample size calculations, and considered outcomes related to vaccine use and safety during pregnancy. The study sites with available laboratory infrastructure also investigated mother-to-child SARS-CoV-2 transmission, as measured by the incidence of detectable SARS-CoV-2 RNA in pregnancy-related fluids and tissues, and followed clinical outcomes of newborns up to 6 weeks after childbirth.

Reporting the procedures related to implementation of large multi-country studies has been encouraged, especially for such cutting-edge clinical and research responses to public health emergencies. Describing experiences and sharing the challenges faced and learned experiences can potentially inform even more efficient and effective research, programme, and policy responses in future outbreaks [4, 5]. This publication summarizes the study development and implementation, its difficulties and challenges, and the key lessons learned throughout the whole process, within different study sites across the world.

## Methods and results

### Coordination and development of the study protocol

In early 2020, WHO recognized that a special focus should be put on the potentially important impact the emerging infectious condition, COVID-19, could have on pregnant women and their infants, as was the case for similar conditions in the past. In March 2020, the WHO Department of Sexual and Reproductive Health (SRH) initiated an open external strategic working group to share information and outline specific proposals for investigating COVID-19 and pregnancy. WHO SRH invited research partners from global networks and based initial proposals on collaborative pregnancy-related research experience from previous outbreaks such as Zika and Ebola [4]. The working group leadership introduced the idea of a generic protocol and sought interest from partners in study implementation. The generic protocol and case report forms (CRFs) were developed between March and May 2020. It was intended for a large international multi-country cohort study that was open to any country; however, funding support was focused mainly on LMICs with newly identified outbreaks. COVID-19 soon showed extensive spread, affecting all continents and countries. WHO determined that recently available emergency funds could be used to support important research to address arising questions about COVID-19. Thus, the pregnancy cohort study protocol became one of the WHO UNITY studies: a series of standardized generic epidemiological investigation protocols focused on COVID-19 [6].

Considering geographic and epidemic diversity, as well as previous experience in performing multi-country studies with WHO, several countries participating in the working group were approached for study implementation. Fifteen countries expressed interest: Argentina, Brazil, Burkina Faso, Chile, Georgia, Ghana, India, Iran, Kenya, Malawi, Myanmar, Pakistan, Philippines, Spain, and Tunisia. However, in five of these countries (Georgia, India, Iran, Malawi and Myanmar) the study could not be fully implemented due to various reasons and therefore the remaining ten countries participated in the WHO cohort study. The generic master protocol received final WHO ethical approval in September 2020, and the first LMIC adapted and approved the protocol, received funding, and began implementing the study in June 2021. Over the course of 2021, as COVID-19 vaccines became increasingly available, including for pregnant women in participating countries, the WHO working group adapted the protocol to include vaccine-related objectives, data collection and analyses. The final set of amendments to the protocol were submitted to WHO ethics in January 2022 and received final approval in June 2022. Although protocols were adapted and vaccine-related data collected in all countries, four of them (Argentina, Brazil, Pakistan, and the Philippines) with a sizeable number of pregnant women receiving COVID-19 vaccines were prioritized and received additional funds to increase the sample size of recruited women for conducting vaccine-related analyses.

### Study design

The study used a prospective cohort design with consecutive recruitment of pregnant or postpartum women (within 48 h of delivery or end of pregnancy). The key exposures evaluated were SARS-CoV-2 infection during pregnancy as determined by virologic and serologic laboratory tests and COVID-19 vaccination status. Women were followed through delivery or end of pregnancy up to 6 weeks postpartum and their neonates up to 4 weeks of life.

### Selection of study sites

This study was intended to be implemented in settings with ongoing SARS-CoV-2 transmission, based on WHO epidemiologic studies or local surveillance data systems. Women could be consecutively recruited in health care facilities, including antenatal care clinics, emergency and labour rooms, COVID-19 testing centres or in the community.

Information on the 10 implementing countries (Argentina, Brazil, Burkina Faso, Chile, Ghana, Kenya, Pakistan, Philippines, Spain and Tunisia) and 43 participating centres is detailed in Table 1. Each of these countries had a national coordinating centre, which identified and approached other centres in the country to be joined in a network for implementing the study, according to their respective human resources and technical capacity to reach the estimated sample size in a reasonable period of time. The number of study sites in each country varied, ranging from 1 to 10, based on balancing geographic and sociodemographic representation with local research capacities. WHO provided SARS-CoV-2 antigen, reverse transcription polymerase chain reaction (RT-PCR), and serology tests, independent of the approved study budget for each country. This was a positive aspect that influenced the willingness of facilities to participate in the study, particularly given the context of the pandemic at the time of study implementation. Serial serologic testing was chosen to detect asymptomatic or mild infection; early in the pandemic, positive results were thought to reflect relatively recent infection. Rapid antigen or RT-PCR testing was collected at enrolment and with any symptoms during follow up. It was pivotal to receive tests with a long expiration date; since for practical reasons, serologic testing was typically conducted on stored samples, which were often transported to a main laboratory or testing facility and held for testing in large batches.

**Table 1:** Study sites in the participating countries, sample size estimates and number of women enrolled

Country	Study sites	Sample size		Enrolment		Remarks
		Target	Actual	Started	Ended	
Argentina	Two maternity hospitals One private and one public Two Argentina regions	1,680	283	Jan/2022	Apr/2023	Enrolment during pregnancy and postpartum (1,1% in the 2º trimester, 61.5% in the 3º trimester and 37.5% in the postpartum period)
Brazil	Nine Maternity hospitals Six Public, one private, and two mixed Four Brazilian regions	2,902	3,109	Aug/2021	Dec/2022	Enrolment during pregnancy and postpartum (11% in the 1º trimester, 25.5% in the 2º trimester, 27.0% in the 3º trimester and 36.5% in the postpartum period)
Burkina Faso	Ten Public Health facilities One Burkina Faso region	1,652	1,785	Feb/2022	Nov/2022	Only enrolment during pregnancy: complete by trimester
Chile	Two Maternity hospitals Two Chilean regions	2,318	288	Mar/2023	Oct/2023	Only enrolment during postpartum
Ghana	Five Public Health facilities One Ghanaian region	1,435	1,444	Jun/2021	Oct/2021	Only enrolment during pregnancy: 1% of women enrolled in first trimester, 16% in second trimester, 83% in third trimester
Kenya	Two Maternity Hospitals One public and one private One Kenian region	1,710	1,717	Jun/2021	Nov/2022	Around 95% enrolment during pregnancy (8% first trimester, 23.7% second and 68.3% third trimester)
Pakistan	Six health facilities Five public and one private Five Pakistan regions	5,236	5,398	Jun/2021	Sep/2022	Only enrolment during pregnancy: 10.9% first trimester, 42.7% second and 46.4% third trimester
Philippines	Five public hospitals One Philippines region	820	733	Sep/2021	Sep/2022	Enrolment mostly in the late 3rd trimester Around enrolment during pregnancy (0.41% first trimester, 0% second and 99.59% third trimester)
Spain	One public maternity hospital	120	55	Jan/2021	Feb/2022	
Tunisia	One referral public maternity hospital	877	1,195	Jul/2021	Dec/2022	Most included in third trimester
Total 10 countries	43 health facilities	18,750	16,007	Jan/2021	Oct/2023	

**Fig. 1:**
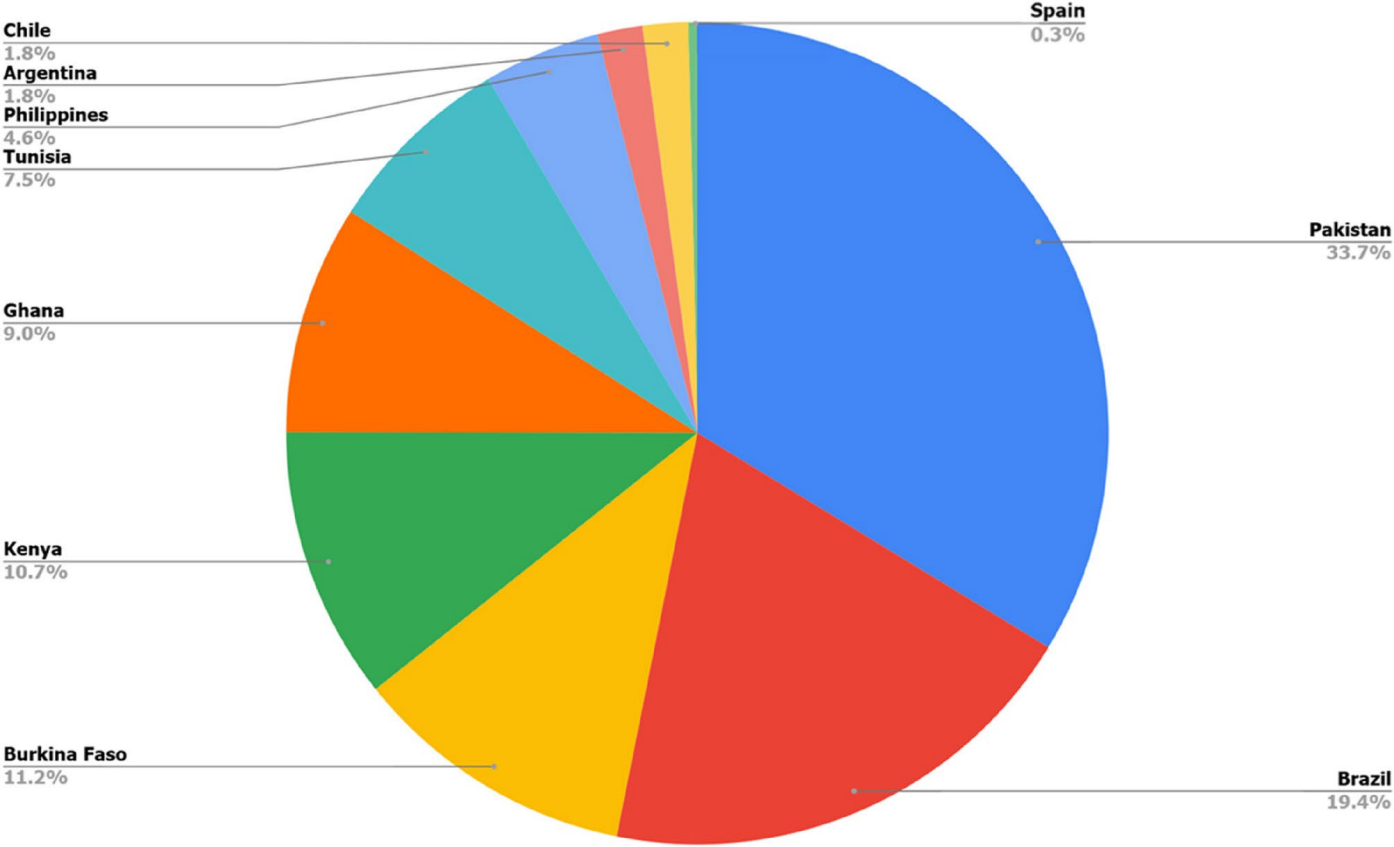
Sample size contribution of the sites to the overall number of enrolled women (*n* = 16,007)

Study sample size calculations in individual countries considered national epidemic circumstances, estimates of main adverse maternal and neonatal health outcomes in each setting, and assessments of exposure to SARS-CoV-2 and vaccination status. Estimated sample sizes ranged from 120 to 5236. Overall, a total of 16,007women were recruited across countries (Fig. 1).

Study sites started recruitment at different time points, depending on local ethical approvals, availability of funding, shipping of test kits, training, and infrastructure preparedness. Enrolment rate and time needed to achieve the estimated sample size also varied greatly (Table 1). The protocol allowed enrolment during antenatal care or up to two days postpartum. Each facility determined the best approach (Table 1). The central WHO coordinating group, however, encouraged early enrolment during antenatal care.

### Staff recruitment and training

There were centrally produced standard operating procedures (SOPs) and on-line meetings for overall training. Each participating country/setting could implement individual strategies and organise their own training plans, to consider local rules/clinical guidelines, follow-up and availability for biological sample collection. Webinars (videos) and written data collection SOPs for CRF completion, including laboratory procedures, sample collection, processing and storage were performed. All these materials were internally developed by the researchers participating in the study. The training program included: (1) screening procedures– identifying, approaching and inviting eligible women, applying informed consent forms and related CRFs; (2) enrolment procedures, including the collection of data and biological samples for laboratory testing; (3) exposure status classification and group allocation; (4) tools for improving follow-up (e.g., “Google calendar”, text messages/WhatsApp for contacting participants, scheduling visits, and completing required information) and accessing study material; (5) procedures related to data quality and good clinical practices (e.g., checklists, data verification, storage of informed consent forms and CRFs); (6) the OpenClinica^®^ platform system for data entry, checking and cleaning. Most countries developed an initial training programme with members of each facility and implemented regular online meetings to ascertain standardization of data collection, progress and quality control. WHO also hosted weekly online meetings (separately by country and/or region) to follow up on implementation of country-specific study protocols, and to review any issues or concerns of the sites from the time of recruitment through delivery and postpartum follow-up. In addition, regular joint “COVID-19 and Pregnancy Cohort Study Implementers Meetings” were organized with all implementing country teams, the WHO study coordination team in Geneva and WHO Regional and Country Office counterparts, to share knowledge, experiences and suggestions. At each joint meeting, a standardized power point presentation template and outline was used for each country to show the study sites, research team, recruitment and data collection status, challenges and strategies to overcome difficulties.

### Monitoring

The WHO coordinating team conducted on-line remote monitoring using weekly meetings with each country and continuous checking of data entry and recruitment numbers. Each country also implemented their own monitoring strategies and data checking.

In some countries, scheduled monitoring visits were also key to ascertaining adequate assistance in data collection, protocol adherence, and consistency assessment of the study in each centre. Visits of researchers from national coordinating centres followed a standard process to monitor study procedures, including completion and storage of informed consent forms and paper CRFs, and evaluation of the infrastructure at each participating site for adequate study implementation. WHO had standard procedures for surveillance of data entry in the OpenClinica^®^ platform, mainly related to providing reminders for delays in entering data, checking internal consistency and generating error lists for each country to resolve. The original plan also included monitoring visits from WHO coordinating staff to all participating countries; however, this was compromised by pandemic-related limitations to international travel. Therefore, Brazil, Burkina Faso and Pakistan (those with the highest overall number of participants) were the only countries to receive such monitoring visits. Nevertheless, special focus was given during multiple joint PI meetings and individual site visits to understand challenges faced by low recruitment sites, with proposal of solution strategies and interventions.

In most countries/settings, investigators also engaged participating institutions with presentation of the study in Department meetings and individually to staff involved in healthcare, especially in antenatal care facilities and during local monitoring visits. Supporting material (posters, folders, QR codes with information on COVID-19 and the project) were also implemented in different settings. These were successful interventions in high recruiting centres.

### Participant recruitment and follow-up

All pregnant women, including minors, and those who delivered or whose pregnancies ended within the previous 48 h were eligible for study enrolment, regardless of SARS-CoV-2 testing results, underlying comorbidities, or COVID-19 vaccination status. Pregnant women who were not available for follow-up (i.e., those planning to deliver in a different city or facility), non-pregnant women, and pregnant women who were incapable of providing informed consent or assent were excluded.

Written informed consent was obtained at enrolment by trained research assistants. Each study site had a specific consent form, listing the exact procedures considered (including SARS-CoV-2 testing, biological samples to be collected, and follow-up), which was approved by the WHO Ethics Review Committee (ERC) and respective national/local IRBs.

**Fig. 2:**
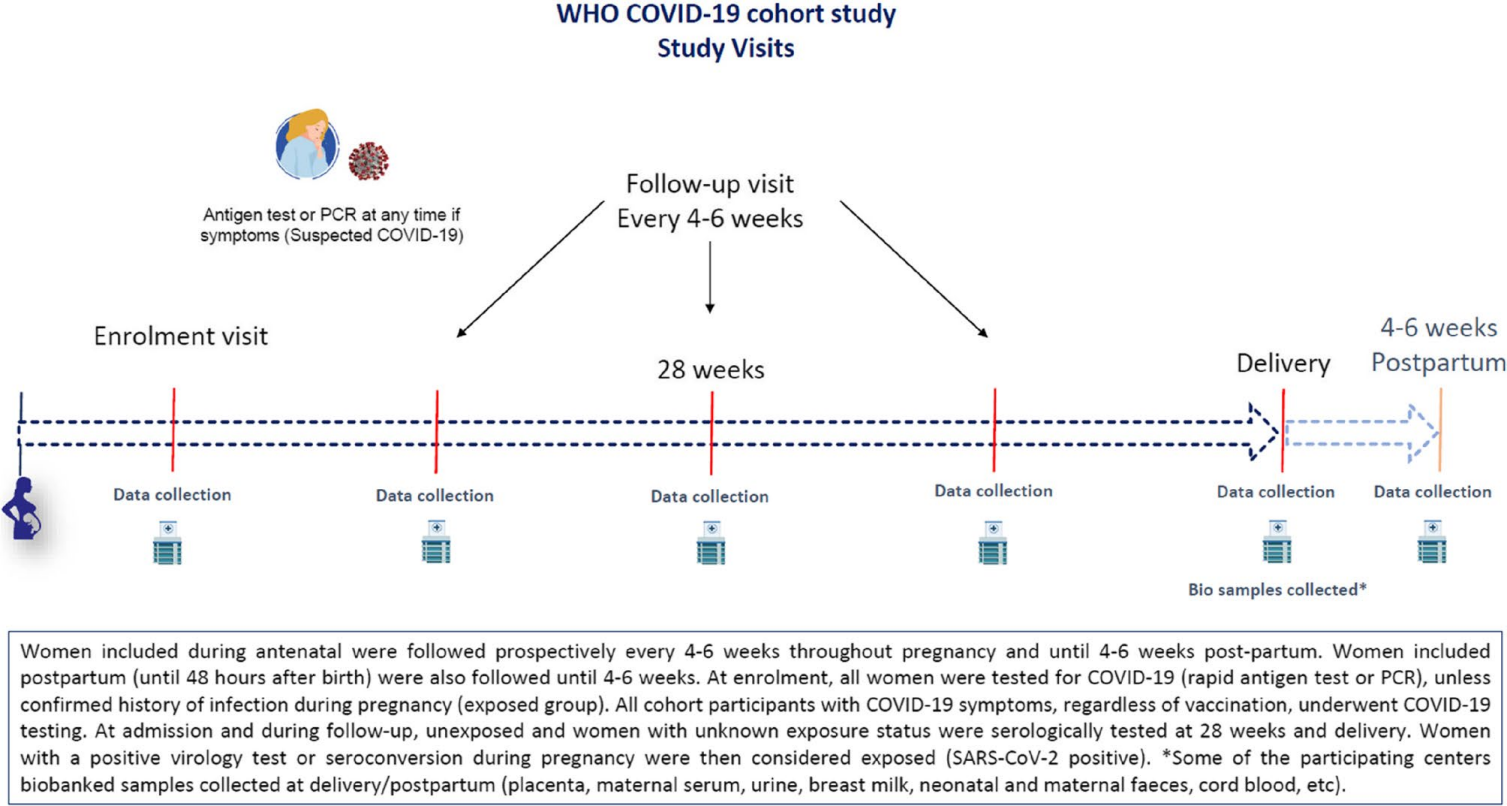
Study procedures, follow-up and considered set points for data and sample collection

Women enrolled during the antenatal period (at any gestational age) were followed prospectively, according to national guidelines on antenatal care (at least once every 4–6 weeks) during pregnancy, childbirth and the postpartum period of 6 weeks (Fig. 2). Women enrolled in the 48 h after delivery were followed until 4–6 weeks postpartum. At enrolment, all women had virologic testing for SARS-CoV-2 (with either rapid antigen test or RT-PCR and serologic testing, unless they already had a documented laboratory-confirmed infection during pregnancy. All cohort participants with COVID-19 symptoms underwent SARS-CoV-2 RT-PCR or antigen testing at any point in the study. Those with negative serologic tests had the tests repeated during follow-up and at delivery. All serology test kits (Wantai^®^ for unvaccinated, and Platelia^®^ if vaccinated) were directly provided to participating countries by WHO and performed in appropriate laboratories with certified capacity. Due to local import procedures, some countries/centres experienced challenges and delays in receiving test kits. Women with a positive virologic test or seroconversion were considered SARS-CoV-2-exposed during pregnancy.

Participants were asked about receiving COVID-19 vaccine at enrolment and at each follow-up appointment, and vaccine cards, registries, and medical records were checked for confirmation and dates. Anti-nucleocapsid (N) serologic tests (Platelia) were used in those with previous vaccination, to distinguish between natural and vaccine-induced antibodies, as most vaccines only generate anti-spike protein (S) antibodies. However, following use of inactivated vaccines (for instance, Sinovac) available serologic tests cannot distinguish between natural and vaccine-induced immune responses, as these vaccines generate both anti-N and anti-S responses, similar to natural infection. Therefore, women who had received an inactivated COVID-19 vaccine had unknown infection status in the absence of a positive virologic test (antigen and/or RT-PCR). This situation typically occurred in Brazil for instance, where the vast majority of the first cases of pregnant women were vaccinated with Coronavac^®^, at least for the first 4–6 months after vaccination campaigns commenced. Sites were recommended to store serum samples from these women for the potential future development of diagnostic solutions for this situation.

### Vaccine coverage

The availability, types and uptake of COVID-19 vaccines varied greatly over time among different settings. In Brazil, over 95% of participants had received at least one dose of vaccine, while in Kenya 38% of women had been vaccinated, in Burkina Faso 5% and in Ghana, only 1% of enrolled women had been vaccinated. Countries reported the use of Sinopharm, Moderna, SINOVAC, Cansino, Pfizer, PakVac, and AstraZeneca vaccines.

### Ethics

After the generic master protocol went through ethics review in WHO, it needed to be adapted to each country’s conditions, budget and the availability of local resources. The PI and local researchers of each national coordinating centre were responsible for local adaptation and translation of the protocol and study instruments to the local language(s) if other than English. This adapted protocol was then technically reviewed by the Human Reproduction Programme (HRP) Research Project Review Panel (RP2) and once approved, submitted to the respective global or regional WHO-ERC, for instance, for centres placed in the Americas, the Pan American Health Organization (PAHO)-ERC was responsible for this ethical review and approval. The final adapted version of the protocol was also assessed and approved by the local and national IRBs according to national regulations.

Considering the pandemic dynamics, availability of new information and interventions, in particular introduction of COVID-19 vaccines, amendments to the original protocol were needed to adjust objectives, sample sizes and data collection forms. The countries’ adaptations to the amended version of the WHO generic protocol needed to be resubmitted to the global or regional WHO ERC and then to the local and national IRBs. Timing for these ethical procedures varied among included centres, with amendments along 2022/2023. Some countries and centres experienced delays in obtaining final approvals.

### Main challenges of implementation and lessons learned

#### Time-sensitive implementation

**Fig. 3:**
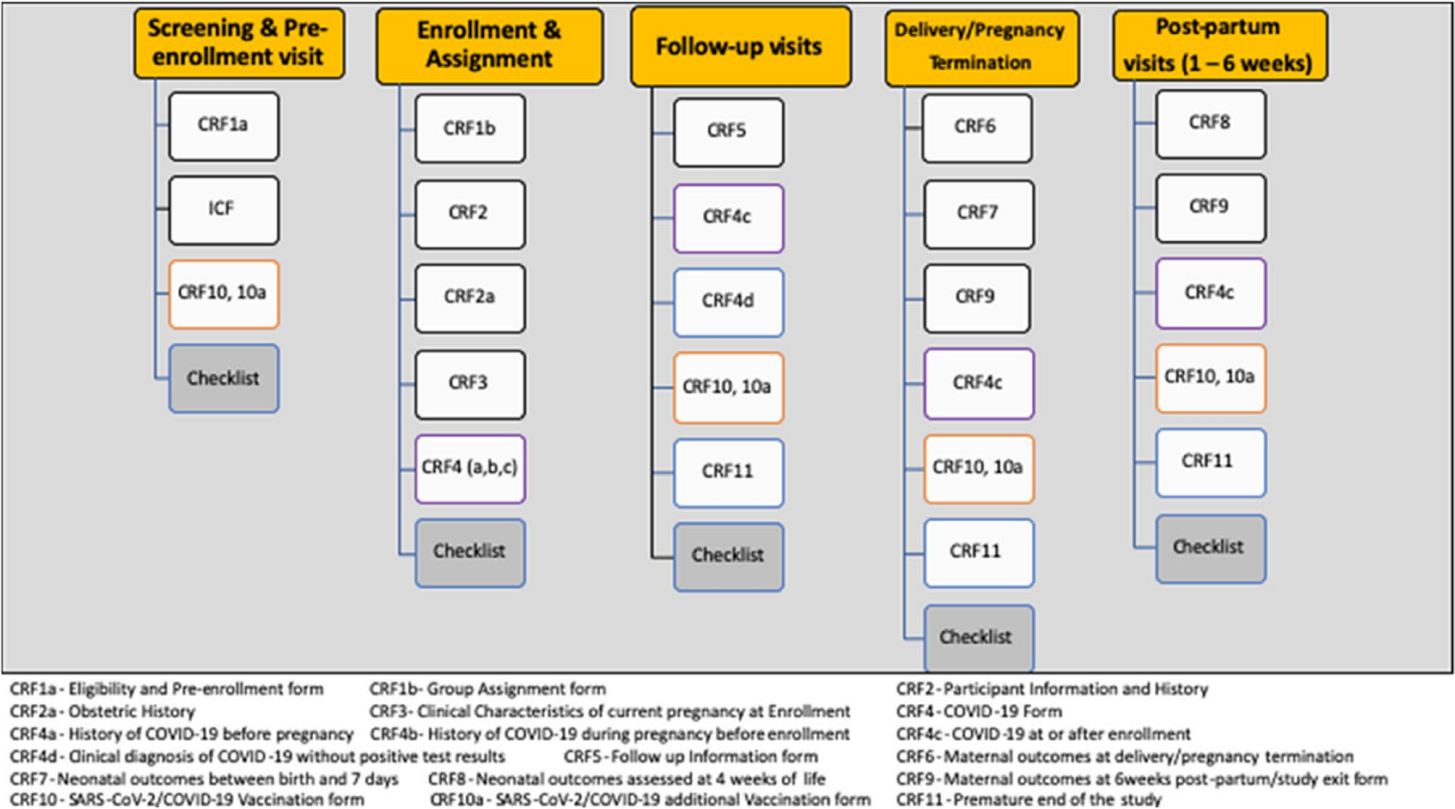
Overview of CRFs that should be completed at various stages of study participation. CRF: case report form; ICF: informed consent form. ^a^Depending on contextual realities, the frequency of follow-up visits may coincide with regular prenatal visit schedule or an alternative schedule. Follow-up visits may be conducted online or via phone to report symptoms or events, as needed

Considering the lack of available data on SARS-CoV-2 infection during pregnancy and postpartum, at the time of project conception and implementation, the decision was made to include a large and comprehensive set of variables on medical and obstetric history, infection, vaccination and maternal, pregnancy, and neonatal outcomes, with an extensive set of 17 case report forms (CRF) comprising 3,169 main variables (Fig. 3).

It was a great challenge to implement such a demanding study with such detailed data and sample collection in LMICs, during the pandemic, with detailed data and sample collection. Initially, clinical practice, availability of testing and implementation of diverse types of vaccines worldwide were evolving and changing rapidly in a very short period of time. These changes throughout the study raised the need for protocol amendments and new sample size calculations to ascertain adequate group allocation and guarantee a sufficient number of participants for achieving the main objectives of the study. Consequently, subsequent resubmissions for ethical review and approval to different boards was needed to catch up with these developments. The mechanisms for ethical approval during the pandemic were not uniform across countries. A good lesson learned and a key recommendation for future global research in similar situations is that a smoother expedited ethics evaluation system is essential in situations of crisis. Ethical review in WHO Regional Offices has been strengthened, and ethical approval from WHO Regional Offices is accepted by WHO HQ, which can save a lot of time.

As part of study implementation, the fundamental role of video conferencing cannot be overemphasized. It was a game changer that enabled international meetings with stakeholders, with PIs of the study, for example to discuss the protocol and amendments, to conduct training, to discuss implementation progress and problems, and to monitor study sites at a time when travel was not recommended or was even cancelled. Without this tool, we would not have been able to do this study.

Other challenges included, for some institutions, unstable internet connection, with the need to install high-speed Wi-Fi terminals. Initially, some also reported difficulties in arranging adequate private space for screening and data collection of eligible women, which was locally resolved in each setting.

In addition, the close coordination between WHO team, the local coordination staff and the field team to identify, discuss and solve emerging issues on an almost daily basis was important for the success of this study. Another learning point was that the final study dataset for analysis will come (relatively) late to make the urgent decisions needed during the pandemic. Results will be published years after the height of the pandemic. Nonetheless, having information on COVID-19 in pregnancy in LMICs, in the post-Omicron era, and having vaccine safety information on non-mRNA vaccines in pregnancy, remains very much needed and relevant.

#### Testing, infrastructure and exposure classification

Implementing SARS-CoV-2 testing at enrolment raised issues for local approvals and patient flow, considering the possibility of a positive result. Local protocols to ascertain safe follow-up and reporting were implemented for such cases. Follow-up serologic testing and sample collection at childbirth were also challenging and unfortunately, many sites were unable to collect these data, especially in settings with antenatal care outpatient clinics that did not refer to the same facility for childbirth. Some centres implemented messaging and communication with patients and offered reimbursement for attending the postpartum evaluation. For future experiences, special interventions to ascertain follow-up are necessary. The increased use of telemedicine and virtual communication might be of use and should be further tested.

Classifying women as confirmed exposed or unexposed to SARS-CoV-2 during pregnancy depending upon serology testing and vaccine type, with ambiguities in categorizing such exposure and its timing, was a key issue.

With advances in vaccination programs in several sites during sample collection, it became increasingly difficult to reach the initially planned sample size for unvaccinated women, e.g., in Tunisia, Brazil, and Argentina, because the uptake of vaccination was high among these populations. Nevertheless, the multicentre approach helped reach target sample sizes for pooled analyses across countries.

Most sites experienced delays in procurement of supplies and equipment for study implementation as well as delays in receiving serology tests. The test kits were purchased and distributed by WHO, which required complex import approvals in some countries and product registration in others. Platelia tests, for example, are not registered in Argentina and it was not possible (during the entire duration of the study), to overcome the bureaucratic procedures for their registration and this led to ending the study before reaching their target sample size. In addition, the short serology expiry dates after these long procedures, resulted in restarting the whole process in some countries.

An important step for the success of the study implementation was availability of an initial readiness budget supporting the costs of necessary laboratory equipment, supplies, and personal protective equipment (PPE) according to the specific needs of each participating country and facilities depending on their structure and resources availability. This helped in building the capacity of the participating health facilities for going ahead with all planned procedures. Availability of funds and needs fluctuated during the pandemic, making this challenging. Additional funds had to be procured to increase the sample sizes. We were fortunate donors stepped in to do this.

A key positive lesson to be highlighted is the importance of a generic protocol allowing standardized implementation of the cohort study and harmonized data collection across multiple different countries. However, there is a need to shorten the data collection forms to the core essential variables. This experience also reinforces the importance of defining those essential variables up front for the next outbreak. Implementing a very detailed and comprehensive questionnaire can be a challenge in itself. We generated a huge amount of information, perhaps more than we are able to analyse and use in a short period of time. This point should be seriously considered in the next generic protocol for a similar purpose. Nonetheless, a special effort is ongoing to conduct ancillary global and country-specific analyses using many of the hundreds of variables collected. In addition, our experience can inform recent initiatives to identify research needs and strategies for evaluating pregnant women affected by future disease outbreaks in a standardized and coordinated manner [7].

It also became evident that government interest should be stimulated for developing research-friendly infrastructure in large public-sector and private-sector hospitals. This would include teams of doctors, nurses and staff prepared for facilitating research during outbreaks, with skills in data collection, data entry, data quality checks, tracking participants and maintaining medical records of enrolled women in such research studies.

#### Specimen processing and laboratory testing

Strategies and SOPs for sample collection, test procedures, and follow-up were developed within each setting to enable virologic testing for SARS-CoV-2 at enrolment and serologic testing during the study and at childbirth. The facilities with capacity for additional biological sample collection and storage (including having a biorepository or biobank already approved) were also supported to include samples such as maternal blood, amniotic fluid, cord blood, placenta, urine, faeces, and breastmilk. Nevertheless, even in places with high technical capacity, challenges occurred, especially because early in the pandemic, many biobanks did not allow for storage and processing of COVID-19 specimens.

Some countries faced the challenge of transporting samples to a testing centre or referral laboratory, either for serology testing or storage. In some situations, even transporting kits to other research facilities was a challenge. In Brazil for example, there was a shortage of dry ice for a few months because national implementation of the vaccination programme used all available stocks. These challenges were individually solved in each country, according to local health system infrastructure and availability of technical and financial resources and other guidance from the WHO study coordination team. In Ghana for example, transportation of specimens from the study sites was aligned with clinic schedules and client loads to facilitate coordination. This was supported through the focal coordinators at the sites.

#### Refusal rate and interventions implemented

Refusal rates (choosing not to enrol during the screening process) greatly varied among study sites. Reports of these data were regularly monitored, and specific strategies and interventions were planned by the PIs and WHO in response. While countries like Ghana, Chile and Spain had virtually no refusal (0%), others, such as Burkina Faso and Kenya experienced, respectively, 37.1% and 38.5% rates of refusal.

Some of the reasons stated for refusal were: hesitancy regarding sample collection, e.g., use of nasal swabs for the antigen test or needle sticks while drawing blood; cultural barriers related to collection of biological samples such as maternal milk; family discouragement; lack of interest in research; fear of contracting the virus from staying longer in the hospital; fear of being isolated post diagnosis and concerns about stigma; and participation in other studies.

Several interventions to address the issue of participant refusal were implemented and experiences shared across settings. For example, in countries like Ghana where acceptance was high, pregnant women reported a desire to protect their unborn babies from the virus as motivation for participation in the study. This informed communication strategies in other countries, especially where refusal was high, to increase acceptance rates. Staff provided reassuring, positive communication when informing women about the study, emphasizing the importance of knowing their own or their baby’s COVID-19 status. Potential benefits of participating in the study were explained, including how the study could help identify early & asymptomatic cases of COVID-19 to provide early and appropriate care. Furthermore, all facility staff were informed and engaged regarding the ongoing research to ensure their support, given their central role as primary caregivers. Refusal gradually declined as the study progressed and teams became more experienced. In order to deeply understand the reasons responsible for higher refusal rates, some settings (like Kenya) even implemented parallel study projects, with a qualitative approach. These results will be relevant for future studies.

#### Training and data quality

The number of research team members, such as health care providers, data managers, and research assistants for data collection and lab procedures, varied across participating facilities according to the setting and number of participants expected. In some countries, the COVID-19 pandemic resulted in rapid shifting and replacement of health care staff, which necessitated repeated training. This increased the workload of the research team, considering the long enrolment period (over a year for some countries) and follow-up.

By the conclusion of this study, almost three years had passed since the protocol was established and the study team assembled. In addition, at times during the pandemic, changes in SARS-CoV-2 variants of concern (VOC) resulted in increased infection rates that caused staff shortages due to medical leave and changing priorities and commitments.

In spite of training and detailed SOPs for entering data into the central OpenClinica^®^ platform, there were large error rates reported by the WHO data coordination centre for almost all countries. Data were collected on almost 20,000 women through around 190,000 CRFs. This huge amount of data and the large number of people involved in data collection and entry resulted in a substantial number of inconsistencies and errors. A well-trained Data Management Centre specific for this study continuously followed data entry for all participating countries, flagging internal inconsistencies in the online system immediately, and also periodically sending each country reports for checking discrepancies and resolving errors. The continuity of this process during the duration of the study facilitated the improvement of data entry clerks’ performance. A set of individual real-time dashboards per country were developed in the Insight module of OpenClinica^®^ system. These dashboards were available to PIs and data managers at country levels with summarized information about key topics (screening, sample size, enrolment, pending data discrepancies and data entry lag) and used for data checking. Finally, an additional challenge was to have all physical forms appropriately scanned and transferred to the WHO repository for quality control and study documentation purposes. In future studies, if feasible in terms of infrastructure, use of physical forms might be reconsidered, to cut down on both paper and workload, with fully on-line data collection.

## Discussion

This multi-country research initiative on COVID-19 and pregnancy represented a significant effort but was feasible, despite the challenges of working in real-time in primarily LMICs with a fast-moving and dynamic pandemic. Rapid variations in the presentation, severity, prevention and management strategies for COVID-19 added to a situation of global confinement unprecedented in the last 100 years, and led to the need to amend protocols, adjust variables and laboratory diagnostic protocols, as well as implementation strategies.

The evolutionary advance of SARS-CoV-2 and emerging VOCs in different settings also placed increased difficulties on study implementation due to research staff infection and medical leave, on top of the increased number of COVID-19 cases and in some scenarios increased severity of disease. In late 2020, four new SARS-CoV-2 VOCs were identified in the United Kingdom (Alpha, PANGO: B.1.1.7), South Africa (Beta, PANGO: B.1.351), Brazil (Gamma, PANGO: P.1) – spreading sharply over Brazilian locations – and India (Delta, PANGO: B.1.617.2) [8]. The Omicron (B.1.1.529) variant was first detected in South Africa in November 2021 and rapidly replaced Delta as the main circulating variant globally, however, associated with less severe disease [9].

All these conditions were difficult to predict. Nevertheless, we have learned for future outbreaks that there is a need for fast and coherent research management and governance, including expedited scientific and ethics review, since it was still lengthy and variable among settings. Templates of core outcomes and variables for generic protocols are needed in advance of pandemics, with sites that would be ready to activate in an outbreak, pending the pathogens involved and the communities affected, as opposed to triggering new processes with each ongoing crisis. In fact, following our study experience, WHO has leaded the definition of a core outcome set for maternal and neonatal health research and surveillance of emerging and ongoing epidemic threats (MNH-EPI-COS): a modified Delphi-based international consensus [7].

Another relevant challenge was maintaining funding across the phases of the pandemic and considering country-specific conditions. The main objective of implementing such a large and consistent study protocol worldwide is to produce timely and relevant responses towards unanswered questions on maternal and perinatal outcomes and enable clinically relevant interventions and healthcare guidelines. Unfortunately, due to the need for amendments involving increased sample sizes, delays in ethical approvals, issues in procuring and importing diagnostic tests, and other challenges, completion of the study took longer than initially expected. One way to optimize timely evidence generation and contribute to larger efforts was authorization by some countries for the secondary use of data in an ongoing prospective individual participant data meta-analysis [3, 10]. For future outbreaks, lessons learned on the quick change in viral infections, either due to variants or because of vaccine introduction, should be considered in preparing the initial study protocol.

A pertinent factor to consider was the constraint imposed by the limitation on in-person meetings during the implementation phase. Nonetheless, a pivotal milestone for nations engaged in the adoption of this protocol was the scheduled final meeting with the country-wide research team. This meeting aimed to assess accomplishments and challenges encountered, as well as to delineate future steps pertinent to the study and its analysis. A few countries have already convened such meetings, with one country even quantifying the environmental impact, considering paper used for data collection during the study’s implementation. In Brazil, paper usage was quantified as equivalent to the consumption of 17 trees. In a commendable initiative, the team proceeded to plant an equivalent number of trees at the University of the Coordinating Centre. There was a final on-line meeting and still planned future meetings for data analysis. While a comprehensive in-person final meeting involving all participating countries remains a prospective endeavour.

## Conclusion

The experience in implementing a large multicentre cohort study of pregnant women in primarily LMIC settings during a pandemic can serve as an example for future research during health emergencies. For COVID-19, this effort not only included collection of epidemiological and clinical data on maternal and perinatal outcomes, but also biological sample collection, viral testing and rigorous follow-up. Our experience demonstrates that producing robust data across disparate LMIC settings during an outbreak can be successfully done. Having pre-established research protocols with a core set of standardized variables, research groups with experience in implementing the required data collection, and expedited ethical review and procurement processes would address some of the challenges we faced and could ensure that data can be analysed in a timely fashion to serve the public health response.

## Data Availability

No datasets were generated or analysed during the current study.
